# A New ELISA for Dermatomyositis Autoantibodies: Rapid Introduction of Autoantigen cDNA to Recombinant Assays for Autoantibody Measurement

**DOI:** 10.1155/2013/856815

**Published:** 2013-12-12

**Authors:** Yoshinao Muro, Kazumitsu Sugiura, Masashi Akiyama

**Affiliations:** Division of Connective Tissue Disease and Autoimmunity, Department of Dermatology, Nagoya University Graduate School of Medicine, 65 Tsurumai-cho, Showa-ku, Nagoya 466-8550, Japan

## Abstract

Advances in immunology, biochemistry, and molecular biology have enabled the development of a number of assays for measuring autoantibodies. ELISA has been widely used, because it can deal with relatively large numbers of serum samples more quickly than other immunologic methods, such as immunoblotting and immunoprecipitation. Recombinant autoantigens, which are generally produced in *E. coli* using the relevant cloned cDNA, are necessary for ELISA. Conventional clinical ELISA tests are limited in their ability to purify proteins free of bacterial contaminants, and the process is labor intensive. We recently developed new ELISA tests that utilize simple *in vitro* transcription and translation labeling of autoantigens in order to measure dermatomyositis- (DM-) specific autoantibodies, including autoantibodies to Mi-2, MDA5, NXP-2, TIF1-**α**, and TIF1-**γ**. This method may allow for the rapid conversion of cDNAs to a chemiluminescent ELISA to detect autoantibodies that are found not only in DM but also in other autoimmune diseases.

## 1. Introduction

Idiopathic inflammatory myopathies (IIMs) are a group of systemic autoimmune diseases that include polymyositis (PM), dermatomyositis (DM), and inclusion body myopathies. Several myositis-specific autoantibodies (MSAs) are associated with certain clinical forms of IIM, and they are useful tools for predicting the prognosis [1]. For example, anti-MDA5 antibody-positive patients demonstrate rapidly progressive interstitial lung disease (ILD), and anti-TIF1-*γ* antibody-positive patients are often complicated with cancer. In contrast, anti-Mi-2 antibodies are a serological marker for favorable prognosis in patients with classical DM who present with typical cutaneous manifestation and myositis. Autoantibodies to TIF1-*γ* are also present in juvenile DM as well as anti-MJ antibodies, and the latter recognize with NXP-2. Autoantibodies in DM tend to be mutually exclusive, thus enabling specific immune responses to differentiate between clinical subsets. It was recently clarified that anti-p155/140 antibodies, which were originally named for the molecular weight of the antigens [2], react to TIF1-*γ* and TIF1-*α*, respectively [3]. It is an exception that anti-TIF1-*α* antibodies appear with two mutually different prognostic markers: anti-TIF1-*γ* antibodies and also anti-Mi-2 antibodies [4].

Laboratories have been using several methods for detecting various autoantibodies: indirect immunofluorescence, immunoprecipitation (IPP), Western blotting (WB), and enzyme-linked immunosorbent assay (ELISA). ELISA-based serologic screening is highly sensitive and efficient, but it requires highly purified recombinant protein. The efficiencies of protein expression, purification, and stability limit the development of a novel ELISA and increase the risk of false-positive antibody detection. At present, many purified recombinant proteins are commercially available; however, full-length recombinant autoantigens are not always available. Moreover, even when they are available, their prices are often very high. Recently, we have developed an ELISA for the detection of antibodies in sera with biotinylated recombinant protein by *in vitro* translation and transcription (TnT) and have detected DM-specific autoantibodies in our DM cohort [4–6]. This review introduces our newly developed ELISA tests, which use recombinant autoantigens to measure DM-specific autoantibodies, mainly autoantibodies to Mi-2, and clarifies the clinical significance of the new assay. This method may allow for the rapid conversion of cDNAs to a chemiluminescent ELISA in order to detect autoantibodies not only in DM but also in other autoimmune diseases.

## 2. ELISA with Commercially Available or In-House Prepared Recombinant DM Autoantigens

Recent works have clarified new DM-specific autoantigens, MDA5, TIF1-*α*/*β*/*γ*, NXP2, and SAE [1]. In some recent studies, ELISAs with some of these commercially available or in-house prepared recombinant autoantigens were used. An ELISA measuring anti-MDA5 antibodies has been used in some works [7–9]. cDNA of MDA5 was cloned by immunoscreening with a patient's sera, and its recombinant protein produced by a baculovirus expression system was used for an ELISA [7]. The analytical sensitivity and specificity of this anti-MDA5 antibody ELISA were 85% and 100%, respectively. Anti-MDA-5 antibody levels measured by this ELISA closely correlated with the severity of skin ulcerations, ILD, and the prognosis of the disease in a Chinese study [8]. In a Japanese study, the median value of the anti-MDA5 antibody titer on admission was higher in patients who later died than in those who survived [9]. The decline index of the anti-MDA5 antibody titer after treatment was lower in the subset of patients who died than in the subset of patients who lived. Sustained high levels of anti-MDA5 antibody were present in the patients who died. In light of these results, anti-MDA5 antibody ELISA is useful for evaluating the response to treatment and the status of ILD in patients with anti-MAD5 antibody-positive DM.

Fujimoto et al. used an ELISA with commercially available recombinant TIF1*γ* and TIF1*α* to investigate longitudinal changes in serum antibody titers [3]. After treatment, the titer of anti-TIF-1*γ* antibodies decreased in all 8 patients, while the titer of anti-TIF-1*α* antibodies did not always decrease. The pathological significance of the titers of TIF1-*γ*/*α* needs further investigation.

Satoh et al. used commercially available recombinant TIF1-*α*/*β*/*γ* in an ELISA [11]. They confirmed the presence of these autoantibodies by using IPP-WB, antigen-capture ELISA, and ELISA with recombinants. The results of the ELISA with recombinants were consistent with the results shown by other immunological methods.

We also tried to perform an ELISA using commercially available recombinant SAE1 [12]. Anti-SAE antibodies were screened for 110 patients with DM, and 2 patients were found to have anti-SAE antibodies. Although anti-SAE autoantibodies also react to another subunit, SAE2 [13], an ELISA with recombinant SAE2 protein has not been reported.

## 3. Recombinant Protein Produced by *In Vitro* Translation and Transcription

Many studies have investigated autoantibodies by using recombinant protein produced by TnT. For example, in studies on cDNA cloning of autoantigens, this eukaryotic expression system, which often uses rabbit reticulocyte lysate, has been utilized in order to confirm whether patient's sera react to candidate clone's product and whether the clone product's mobility on SDS-PAGE is the same as the mobility of the endogenous cellular antigen [14–16]. Recombinant proteins produced by TnT are generally labeled with ^35^S-methionine. The productive efficiency is theoretically influenced by the presence of the Kozak's consensus sequence around the AUG initiation codon and the numbers of methionine residues. Recent commercial kits for TnT contain all the necessary materials, except for highly purified DNA, to produce recombinants. The recombinant protein can be used for IPP without any pretreatment, since it is generally produced in soluble form.

To eliminate the need for radioactive materials, commercial products for biotin-labeled recombinants are also available. This labeling utilizes precharged *E. coli* lysine tRNAs, which are chemically biotinylated at the *ε*-aminogroup. Biotinylated proteins can be detected by the binding of streptavidin-alkaline phosphatase or streptavidin-horseradish peroxidase using a colorimetric or chemiluminescent detection system. Although the presence of biotinylated lysines may affect the antigenic structure of the modified protein, in our experience, the detection of anti-DFS70 antibodies using IPP with the biotinylated recombinant protein is largely consistent with their detection by WB with bacterial recombinant protein [17]. Detections of anti-MDA-5 and anti-TIF1-*γ* antibodies using IPP with the biotinylated recombinant protein are also closely consistent with their detection by the standard IPP with radio-labeled cellular extract [18].

## 4. ELISA with Biotinylated Recombinant Protein

**Figure 1:**
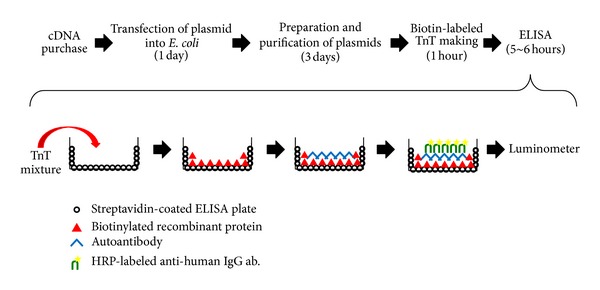
ELISA development using biotinylated recombinant protein. The process flow summarizes the method of ELISA construction, from obtaining the cDNA to obtaining the data by luminometer. We perform phenol/chloroform treatments twice to inhibit RNase for the plasmid purification.

We applied the above recombinant protein biotinylated* in vitro* TnT system to ELISA. After cDNA inserted into a plasmid vector containing T7 promotor is purchased, it takes up to 10 days to construct an ELISA system for the measurement of autoantibodies (Figure 1). At the first attempt, biotinylated MDA5 recombinants were coated onto commercial ELISA plates to which streptavidin was covalently coupled via a spacer [5]. This procedure also enabled the recombinant protein to be purified from crude lysate. Although this measurement could have been done with a conventional optical system for ELISA, 10 *μ*L of reaction mixture per well was necessary to get a sufficiently positive signal, at the cost of ~$5/well. If we were able to purchase a commercial purified recombinant protein for less than $100/*μ*g, it would be more cost effective for building an ELISA. To save the cost, we used a microplate luminometer to increase the sensitivity, thereby reducing the amount of biotinylated recombinant protein required for the assays [4, 6]. Anti-MDA5 antibody levels obtained using a luminometer had a good correlation with those obtained using spectrophotometer [19]. We were able to reduce the amount of reaction mixture of TnT from 10 *μ*L/well to 1 *μ*L/well. Even if the cost of streptavidin-coated plate and highly sensitive chemiluminescence substrate are added, the per-well cost will still be only around $1.

## 5. Anti-Mi-2 Antibodies Measured by Our ELISA

Anti-Mi-2 antibodies were the first to be identified as DM-specific marker autoantibodies [20]. Patients with DM carrying these antibodies often show classic DM skin lesions and favorable prognosis [1]. The target macromolecular protein is the Mi-2/NuRD complex, which is involved in multiple transcriptional regulatory processes [21]. Anti-Mi-2 autoantibodies mainly target Mi-2*α*/*β* that are around 240 kDa [22]. Previous epitope-mapping studies showed multiple antigenic regions on the polypeptides of Mi-2*β* [23]. Even the most antigenic fragment was reactive to less than 60% of anti-Mi-2-positive samples. ELISA kits for anti-Mi-2 are available from one company (Thermo Fisher Scientific Inc.), and these were validated by a single study [24]. Since full-length recombinant proteins of Mi-2*α*/*β* are not available in Japan, we tried to construct an ELISA for the measurement of anti-Mi-2 antibodies.

The full-length Mi-2*β* cDNA clone [25] was a kind gift from Drs. Kato and Takahashi at Nagoya University. The plasmid harboring this clone contains the T7 promotor and the HA-tag and V5/His-tag at the N-terminus and C-terminus, respectively. Biotinylated recombinant protein was produced from the cDNA, using the TnT T7 Quick Coupled Transcription/Translation System (Promega, Madison, USA) according to our protocol [4]. Nunc Immobilizer Streptavidin Plates (Thermo Scientific Nunc, Roskilde, Denmark) were prewashed 3 times with PBS containing 0.05% Tween 20 (T-PBS), coated with TnT product diluted with T-PBS (50 *μ*L/well), and incubated for 1 hour at room temperature. After 3 washes with T-PBS, the wells were blocked with 200 *μ*L of a blocking buffer of 0.5% bovine serum albumin (Wako, Osaka, Japan) in T-PBS for 1 hour. Uncoated wells were used to measure the background levels for each sample. Sample sera diluted with blocking buffer (50 *μ*L/well) were incubated for 1 hour at room temperature, followed by incubation with antihuman IgG antibody conjugated with HRP (Dako, Glostrup, Denmark) (50 *μ*L/well) at 1 : 30,000 dilution. After incubation for 1 hour at room temperature, the plates were washed and incubated with SuperSignal ELISA Femto Maximum Sensitivity Substrate (Thermo Scientific Pierce, Rockford, USA) (50 *μ*L/well) as the substrate. Then, relative luminescence unit (RLU) was determined using the GloMax-Multi Detection System (Promega). Each serum sample was tested in duplicate, and the mean RLU-subtracted background was used for data analysis. The high-level anti-Mi-2 antibody-positive serum serially diluted to 1 : 5, starting from 1 : 500, was run as standard. Units correlated with the antibody titers of antibodies: 1 : 500 dilution, 625 units; 1 : 2,500 dilution, 125 units; 1 : 12,500 dilution, 25 units; 1 : 62,500 dilution, 5 units; 1 : 312,500 dilution, 1 unit.

From the serum bank of the Department of Dermatology, Nagoya University Hospital, we screened anti-Mi-2*β* antibodies in sera from 124 Japanese patients with DM (including 13 with juvenile DM, 39 with clinically amyopathic DM, and 19 with cancer-associated DM), in which 108 serum samples had been used in our previous study [4]. Five sera from patients with DM immunoprecipitated the biotinylated recombinant Mi-2*β* by IPP [4], and these had been confirmed to have anti-Mi-2 antibodies by IPP-WB by using anti-Mi-2*α* monoclonal antibody [4]. Twenty healthy individuals were assessed as normal controls. This study was approved by the Ethics Committee of the Nagoya University Graduate School of Medicine and conducted in accordance with the Declaration of Helsinki.

**Figure 2:**
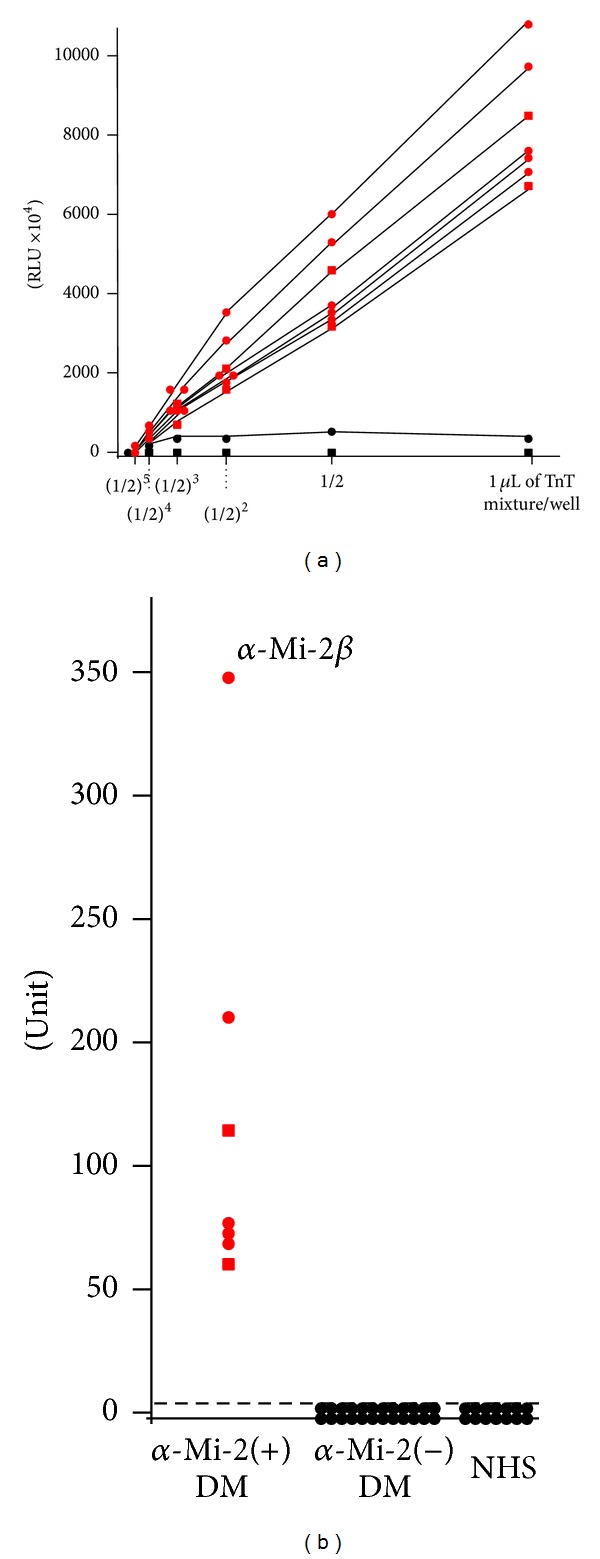
ELISA using biotinylated recombinant Mi-2*β* protein. (a) Serial dilution of biotinylated *in vitro* translation and transcription product for ELISA. Red circles: anti-Mi-2 positive sera defined in our previous analysis. Red squares: newly identified anti-Mi-2 positive sera. Black circles: serum from patients with DM having high background. Black squares: healthy individual serum. Recombinant protein was diluted with T-PBS to 50 *μ*L of the final volume per well. Serum dilution was 1 : 1,000. RLU = relative luminescence unit. (b) Measurement of anti-Mi-2*β* antibodies in 128 serum samples from patients with DM or 20 healthy control subjects (NHS). We used the 0.5 *μ*L/well of TnT mixture and patient serum samples diluted to 1 : 1000 for measuring all samples. Antibody units were calculated from the RLU using a standard curve obtained from serial concentrations of a serum sample containing a high titer of the anti-Mi-2*β* antibody. The broken line indicates the cut-off value (0.53 units).

All 5 anti-Mi-2 positive sera identified as such by our previous study were also reactive to the recombinant in ELISA (Figure 2(a)). Using one positive serum with the high titer as the ELISA standard, all serum samples from DM patients and healthy individuals were investigated by ELISA, for which 0.5 *μ*L/well of TnT reaction mixture was used. The cut-off level was set at 0.53 units, based on 5 standard deviations (SDs) above the mean value obtained from 20 healthy control sera. Two additional sera, which were not included in the previous study, were newly found to have anti-Mi-2 antibodies. No serum samples from the other 117 patients with DM or from the healthy individuals reached the cut-off level. One serum sample from a patient showed over 3 SDs above the mean value obtained from controls: 0.46. This serum showed no dose dependency for the amount of coated antigen (Figure 2(a)). Moreover, when only reticulocyte lysate-coated wells were used as the background for subtraction instead of uncoated wells being used, this serum unit fell below the 3SDs+mean (data not shown). Twenty ELISA-negative serum samples from DM patients were confirmed to be anti-Mi-2 negative by IPP-WB (data not shown). The clinical profiles of the 7 anti-Mi-2-positive patients are summarized and compared with data of a published Japanese multicenter study [10] in Table 1. Although ages at onset and sex ratios are different between the two studies, anti-Mi-2 positive DM is regarded as being classical DM and having a favorable prognosis without the life-threatening complication of malignancy or ILD [1].

## 6. Advantages and Disadvantages of the New ELISA

The results presented in this study demonstrated that a rapid, specific, sensitive, and quantitative anti-Mi-2-antibody immunoassay can be created by using commercially available *in vitro* TnT kits and precoated ELISA plates. Our newly established ELISA is a simple experimental method that does not require the use of radioisotopes. It is probably applicable for measuring various kinds of autoantibodies.

Standard IPP using cell extract has some limitations for accurate interpretation. Many DM-/PM-specific autoantigens show similar migration patterns on gel electrophoresis: from 100 kDa to 200 kDa. Some antigens may be insufficiently expressed in standard cultured cell lines. Although many commercial measuring kits can be purchased, commercial laboratory kits for new diagnostic autoantibodies are not readily available [26]. Traditional methods that use purified recombinant proteins are labor intensive and may take months to obtain proteins of sufficient purity and optimization prior to ELISA development. This process limits the rapid serologic analysis of novel antigens. Recently, many purified recombinant proteins have become commercially available. However, they are usually expensive, and the various kinds of epitope tags depend on each company. It is often difficult to find commercially available recombinant proteins of large molecule size as full-length proteins. For example, Mi-2 antigens are >200 kDa, and their full-length recombinants are not yet commercially available in Japan. In some countries, various recombinant DM autoantigens, including Mi-2*β*, have recently become commercially available, for example, from Diarect AG (Freiburg, Germany) and SurModics, Inc. (Eden Prairie, MN, USA).

Our assay has several limitations for detecting antibodies. When recombinant proteins are prepared by *in vitro* TnT, post-translational modification does not fully occur. Some autoantibodies are regarded as recognizing the post-translational modification of the protein [27]. Autoantibodies to insulinoma-associated protein 2 (IA-2), which are a serological marker of insulin-dependent diabetes mellitus, preferentially react to baculovirus-expressed IA-2 slightly better than *in vitro* translated IA-2 reacts [28, 29]. Autoantibodies to thyrotropin receptor (TSHR) in Graves' disease do not efficiently bind to the TSHR recombinant produced in an *in vitro* TnT system [30, 31]. The above data are probably due to the glycosylation of IA-2 and TSHR, which plays an important role in autoepitopes of these antigens and may occur in baculovirus expression system but not in TnT system.

The expected amounts of recombinant protein derived from the positive control plasmid are in the range of ~300 ng of protein in a standard 50 *μ*L reaction, according to manufacturer's instructions. Protein expression with *in vitro* TnT can vary from batch to batch, and quantification of the resulting protein concentration is challenging. Estimating incorporation levels of biotinylated lysine is more difficult. Although large proteins are sometimes difficult to express as recombinant full product, we succeeded in producing Mi-2*β* by using a TnT system [4]. Recently, we have also succeeded in producing larger autoantigens, for example, envoplakin and periplakin, by using this system [32].

**Figure 3:**
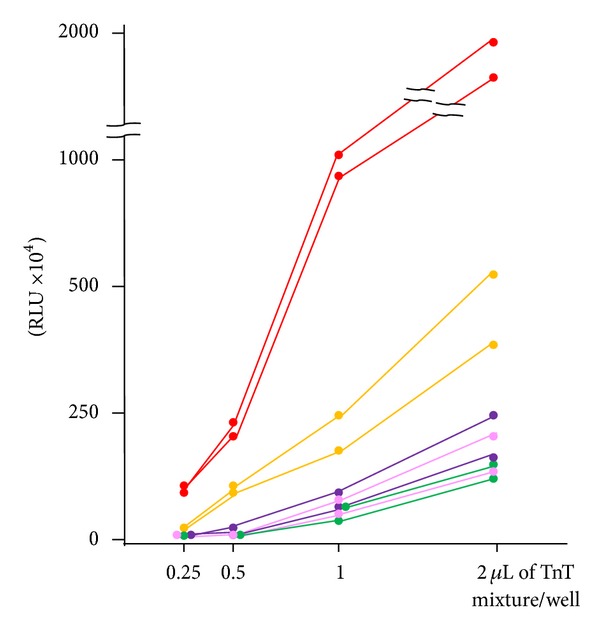
ELISA using biotinylated recombinant proteins of 5 different DM-specific autoantigens. Serial dilution of biotinylated *in vitro* translation and transcription product for ELISA using 2 representative positive sera from DM patients. Serum dilution was 1 : 1,000. Red circles: Mi-2*β*. Yellow circles: MJ (NXP-2). Purple circles: TIF1-*α*. Pink circles: MDA5. Green circles: TIF1-*γ*. RLU = relative luminescence unit.

The biggest potential issue with our system is that the presence of biotinylated lysines may affect epitope recognition by the autoantibodies. We compared our ELISA results of representative positive sera for 5 different DM-marker autoantigens: Mi-2*β*, MJ (NXP2), MDA5, TIF1-*α*, and TIF1-*γ* (Figure 3). Recombinant Mi-2*β* was much more highly reactive than the other antigens. Although the reason is obscure, several possibilities are considered. The ratio of lysine content in Mi-2*β* (9.2%) is the highest among these antigens and, interestingly, the sequence has 3 short lysine stretches consisting of 5 or 6 residues at the N-terminus, which may incorporate biotin-labeling efficiently.

An improved method can be considered that uses TnT recombinants without biotinylation. Tag polypeptide and its ligand for coating tagged proteins, the binding of which is as strong as biotin-streptavidin binding, can be applied, such as Halo tag and its ligand [33]. We plan to investigate whether an ELISA constructed with a cDNA clone inserted into the Halo tag vector containing T7 promotor and with ligand-coated plate improves the reactivities of autoantibodies, including anti-TIF1-*α* and anti-TIF1-*γ* antibodies.

## Figures and Tables

**Table 1:** Comparison of clinical data for patients with anti-Mi-2 antibody in a previous report and in the present study.

	Multicenter study*	This study
Anti-Mi-2 (+) pts., number (%)	9/376 (2.4)	7/124 (5.6)
Age at onset, median (range), y	45 (16–66)	62 (40–73)
Sex, M/F, number	6/3	1/6
Diagnosis, %		
Classical DM	100	100
Clinically amyopathic DM	0	0
Clinical features, %		
Muscle weakness	100	100
Arthritis	11	14
ILD	11	0
Malignancy	0	0
Skin eruptions, %		
Heliotrope rash	67	57
Facial erythema**	56	100
Gottron sign	89	100
Prognosis (alive), %	100	100

*Data from [10]. This cohort includes 7 patients used in this study, all of whom were negative for anti-Mi-2 antibodies. **Facial erythema other than heliotrope rash.

## References

[1] Tansley SL, Betteridge ZE, McHugh NJ (2013). The diagnostic utility of autoantibodies in adult and juvenile myositis. *Current Opinion in Rheumatology*.

[2] Kaji K, Fujimoto M, Hasegawa M (2007). Identification of a novel autoantibody reactive with 155 and 140 kDa nuclear proteins in patients with dermatomyositis: an association with malignancy. *Rheumatology*.

[3] Fujimoto M, Hamaguchi Y, Kaji K (2012). Myositis-specific anti-155/140 autoantibodies target transcription intermediary factor 1 family proteins. *Arthritis and Rheumatism*.

[4] Hoshino K, Muro Y, Sugiura K, Tomita Y, Nakashima R, Mimori T (2010). Anti-MDA5 and anti-TIF1-*γ* antibodies have clinical significance for patients with dermatomyositis. *Rheumatology*.

[5] Muro Y, Sugiura K, Hoshino K, Akiyama M (2012). Disappearance of anti-MDA-5 autoantibodies in clinically amyopathic DM/interstitial lung disease during disease remission. *Rheumatology*.

[6] Ishikawa A, Muro Y, Sugiura K, Akiyama M (2012). Development of an ELISA for detection of autoantibodies to nuclear matrix protein 2. *Rheumatology*.

[7] Sato S, Hoshino K, Satoh T (2009). RNA helicase encoded by melanoma differentiation-associated gene 5 is a major autoantigen in patients with clinically amyopathic dermatomyositis: association with rapidly progressive interstitial lung disease. *Arthritis and Rheumatism*.

[8] Cao H, Pan M, Kang Y (2012). Clinical manifestations of dermatomyositis and clinically amyopathic dermatomyositis patients with positive expression of anti-melanoma differentiation-associated gene 5 antibody. *Arthritis Care and Research*.

[9] Gono T, Sato S, Kawaguchi Y (2012). Anti-MDA5 antibody, ferritin and IL-18 are useful for the evaluation of response to treatment in interstitial lung disease with anti-MDA5 antibody-positive dermatomyositis. *Rheumatology*.

[10] Hamaguchi Y, Kuwana M, Hoshino K (2011). Clinical correlations with dermatomyositis-specific autoantibodies in adult Japanese patients with dermatomyositis: a multicenter cross-sectional study. *Archives of Dermatology*.

[11] Satoh M, Chan JYF, Ross SJ (2012). Autoantibodies to Transcription Intermediary Factor (TIF)1*β* associated with dermatomyositis. *Arthritis Research and Therapy*.

[12] Muro Y, Sugiura K, Akiyama M (2013). Low prevalence of anti-small ubiquitin-like modifier activating enzyme antibodies in dermatomyositis patients. *Autoimmunity*.

[13] Betteridge Z, Gunawardena H, North J, Slinn J, McHugh N (2007). Identification of a novel autoantibody directed against small ubiquitin-like modifier activating enzyme in dermatomyositis. *Arthritis and Rheumatism*.

[14] Ben-Chetrit E, Gandy BJ, Tan EM, Sullivan KF (1989). Isolation and characterization of a cDNA clone encoding the 60-kD component of the human SS-A/Ro ribonucleoprotein autoantigen. *Journal of Clinical Investigation*.

[15] Chan EKL, Imai H, Hamel JC, Tan EM (1991). Human autoantibody to RNA polymerase I transcription factor hUBF. Molecular identity of nucleolus organizer region autoantigen NOR-90 and ribosomal RNA transcription upstream binding factor. *Journal of Experimental Medicine*.

[16] Imai H, Chan EKL, Kiyosawa K, Fu X-D, Tan EM (1993). Novel nuclear autoantigen with splicing factor motifs identified with antibody from hepatocellular carcinoma. *Journal of Clinical Investigation*.

[17] Ogawa Y, Sugiura K, Watanabe A (2004). Autoantigenicity of DFS70 is restricted to the conformational epitope of C-terminal alpha-helical domain. *Journal of Autoimmunity*.

[18] Hoshino K, Muro Y, Sugiura K, Tomita Y, Nakashima R, Mimori T (2010). Anti-MDA5 and anti-TIF1-*γ* antibodies have clinical significance for patients with dermatomyositis. *Rheumatology*.

[19] Muro Y, Sugiura K, Akiyama M (2013). Limitations of a single-point evaluation of anti-MDA5 antibody, ferritin, and IL-18 in predicting the prognosis of interstitial lung disease with anti-MDA5 antibody-positive dermatomyositis. *Clinical Rheumatology*.

[20] Targoff IN, Reichlin M (1985). The association between Mi-2 antibodies and dermatomyositis. *Arthritis and Rheumatism*.

[21] Wang H-B, Zhang Y (2001). Mi2, an auto-antigen for dermatomyositis, is an ATP-dependent nucleosome remodeling factor. *Nucleic Acids Research*.

[22] Seelig HP, Renz M, Targoff IN, Ge Q, Frank MB (1996). Two forms of the major antigenic protein of the dermatomyositis-specific Mi-2 autoantigen. *Arthritis and Rheumatism*.

[23] Hengstman GJD, Vree Egberts WTM, Seelig HP (2006). Clinical characteristics of patients with myositis and autoantibodies to different fragments of the Mi-2*β* antigen. *Annals of the Rheumatic Diseases*.

[24] Parker JC, Bunn CC (2011). Sensitivity of the Phadia EliA connective tissue disease screen for less common disease-specific autoantibodies. *Journal of Clinical Pathology*.

[25] Shimono K, Shimono Y, Shimokata K, Ishiguro N, Takahashi M (2005). Microspherule protein 1, Mi-2*β*, and RET finger protein associate in the nucleolus and up-regulate ribosomal gene transcription. *Journal of Biological Chemistry*.

[26] Muro Y, Sugiura K, Akiyama M (2013). What autoantibody tests should become widely available to help scleroderma diagnosis and management?. *Arthritis Research and Therapy*.

[27] Doyle HA, Mamula MJ (2001). Post-translational protein modifications in antigen recognition and autoimmunity. *Trends in Immunology*.

[28] Lan MS, Wasserfall C, Maclaren NK, Notkins AL (1996). IA-2, a transmembrane protein of the protein tyrosine phosphatase family, is a major autoantigen in insulin-dependent diabetes mellitus. *Proceedings of the National Academy of Sciences of the United States of America*.

[29] Xie H, Deng Y-J, Notkins AL, Lan MS (1998). Expression, characterization, processing and immunogenicity of an insulin-dependent diabetes mellitus autoantigen, IA-2, in Sf-9 cells. *Clinical and Experimental Immunology*.

[30] Prentice L, Sanders JF, Perez M (1997). Thyrotropin (TSH) receptor autoantibodies do not appear to bind to the TSH receptor produced in an in vitro transcription/translation system. *Journal of Clinical Endocrinology and Metabolism*.

[31] Seetharamaiah GS, Kaithamana S, Desai RK, Prabhakar BS (1999). Regulation of thyrotropin receptor protein expression in insect cells. *Journal of Molecular Endocrinology*.

[32] Muro Y, Sugiura K, Shiraki A (2014). Detection of autoantibodies to periplakin and envoplakin in paraneoplastic pemphigus but not idiopathic pulmonary fibrosis using full-length recombinant protein. *Clinca Chimica Acta*.

[33] Urh M, Rosenberg M (2012). HaloTag, a platform technology for protein analysis. *Current Chemical Genomics*.

